# Supplementary figures and images for: High-throughput screening reveals diverse lambdoid prophage inducers
Source: Microbiol Spectr. 2025 Oct 27;13(12):e01707-25. doi: 10.1128/spectrum.01707-25 (PMC12671112; doi:10.1128/spectrum.01707-25)

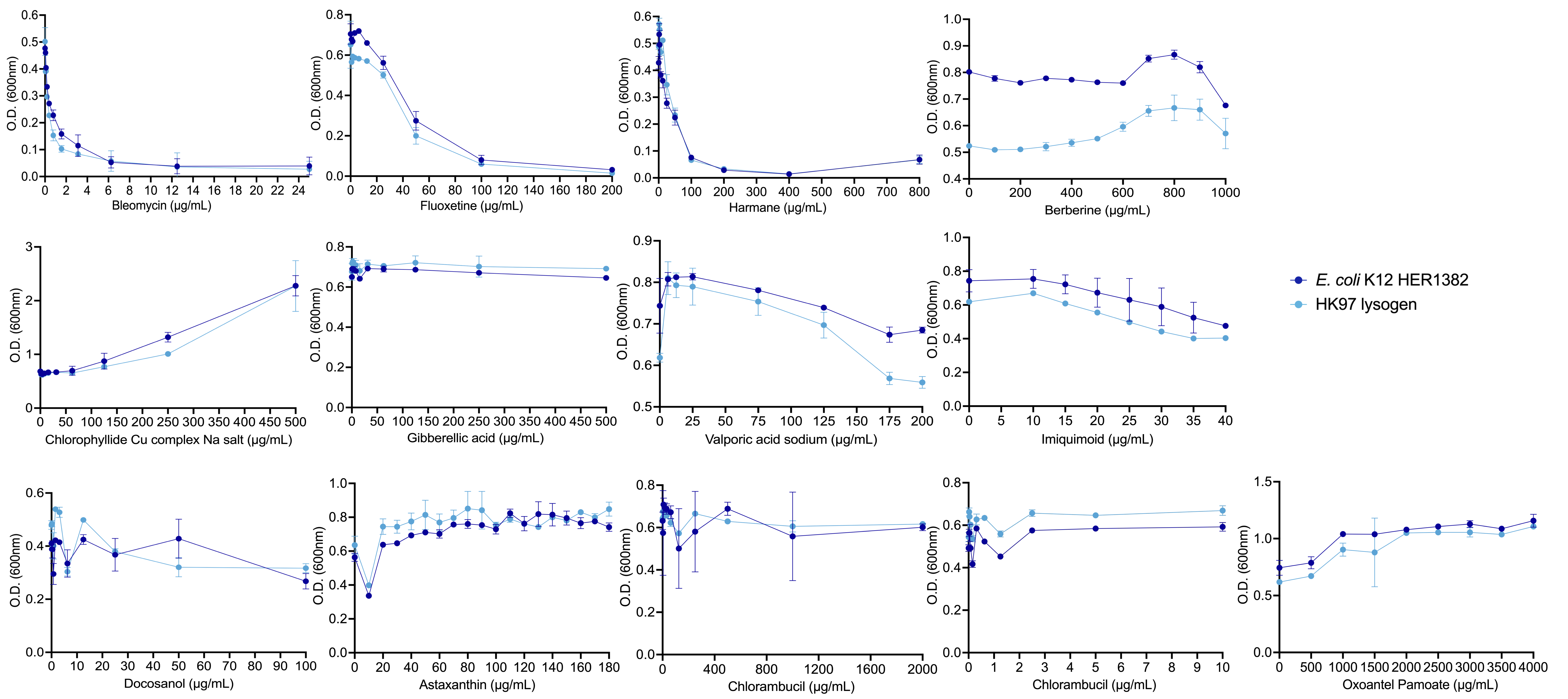

Supplement: Fig. S1 — MIC curves of ordered primary hits. [file spectrum.01707-25-s0002.tif]

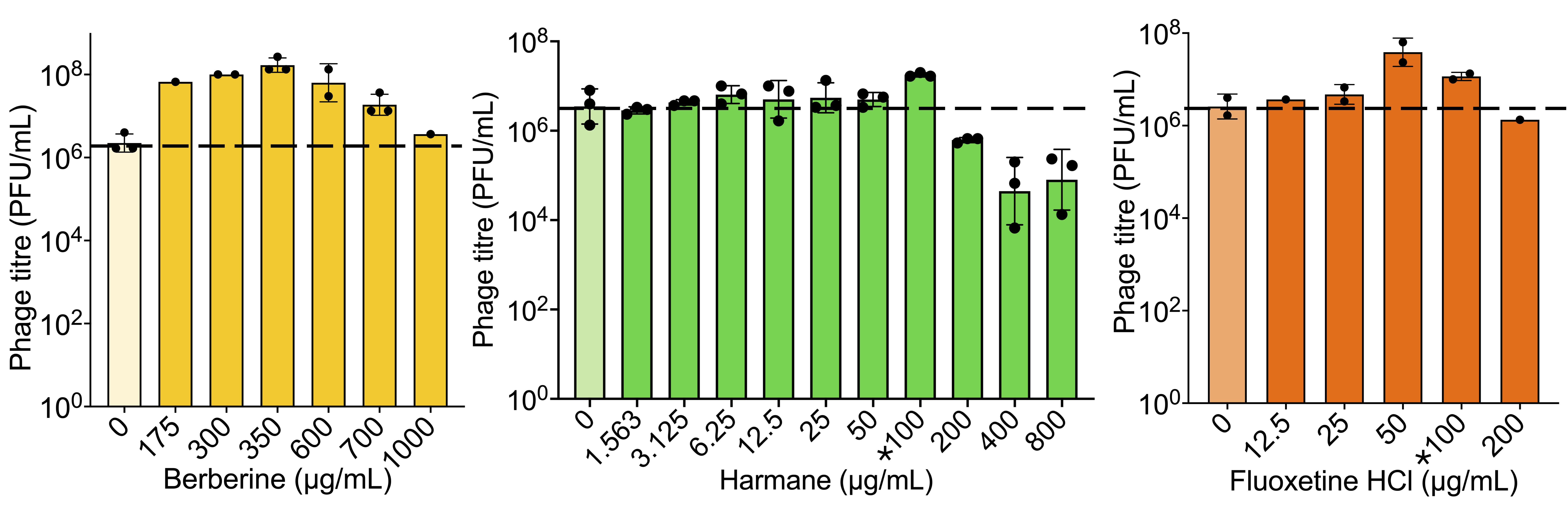

Supplement: Fig. S2 — Berberine, harmane, and fluoxetine HCl have narrow range of prophage induction. [file spectrum.01707-25-s0003.tif]

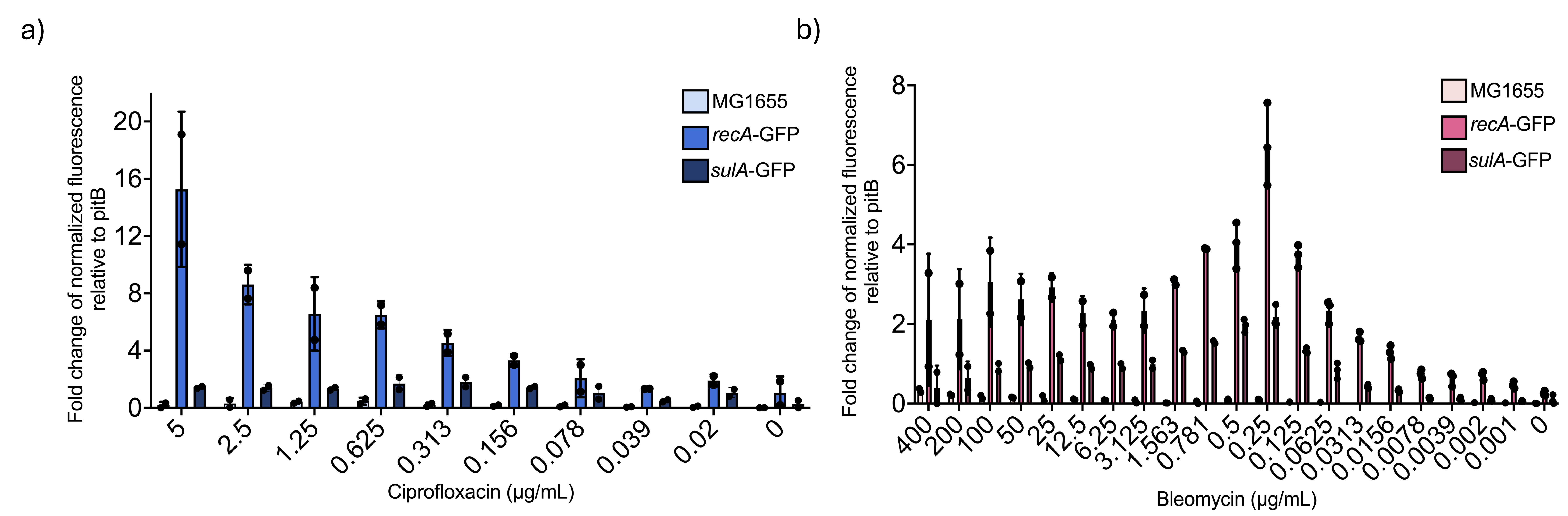

Supplement: Fig. S3 — Exposure to bleomycin results in consistent levels of SOS induction across a concentration range normalized normalized to pitB-GFP. [file spectrum.01707-25-s0004.tif]

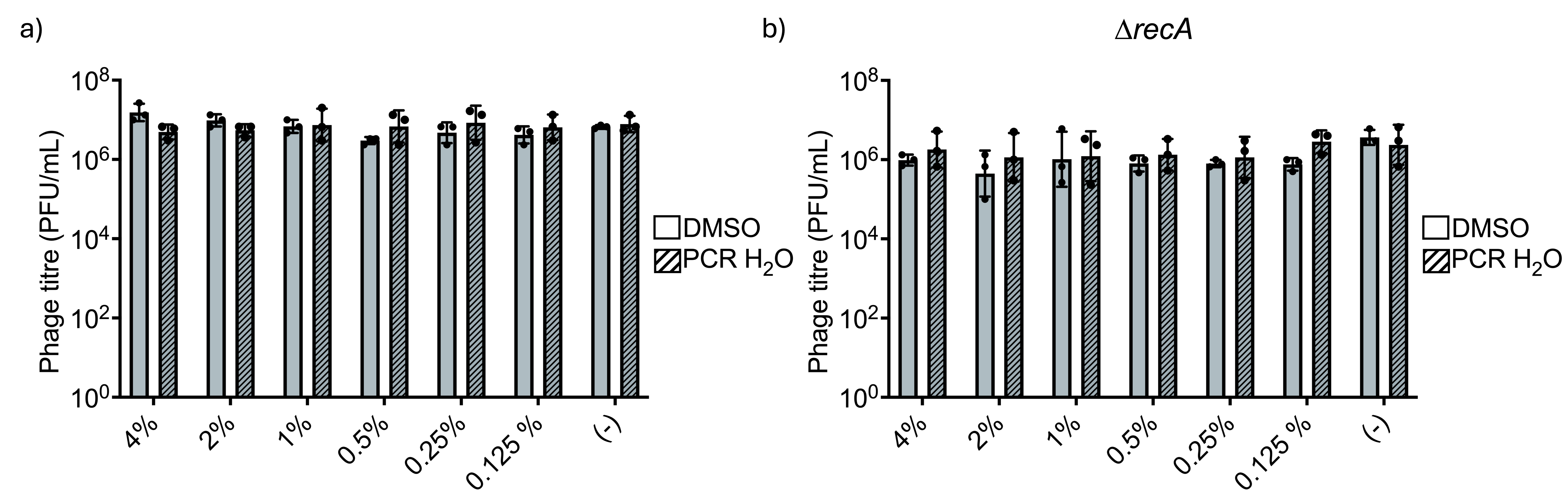

Supplement: Fig. S4 — Solvent used to dissolve bioactive compounds has no effect on induction. [file spectrum.01707-25-s0005.tif]
