# Supplementary material for: High-throughput screening reveals diverse lambdoid prophage inducers
Source: Microbiol Spectr. 2025 Oct 27;13(12):e01707-25. doi: 10.1128/spectrum.01707-25 (PMC12671112; doi:10.1128/spectrum.01707-25)
Supplement: Supplemental material — Legends for Fig. S1 to S4; Tables S1 to S3. [file spectrum.01707-25-s0006.docx]

**Supplementary**

**Figure S1: MIC curves of ordered primary hits.** Endpoint (18 h) Optical density reads of *E. coli* K12 HER1382 and HK97 lysogen either alone or challenged with 13 different primary hits across a range of concentrations, averaged across 1-3 biological replicates. Error bars represent SD.

**Figure S2: Berberine, Harmane and Fluoxetine HCl have narrow range of prophage induction.** Phage quantification of HK97 lysogen either alone, or challenged with Berberine, Harmane, or Fluoxetine across a range of concentrations, averaged across 1-3 biological replicates with each black dot representing a replicate. * Denotes MIC. Dotted line represents baseline induction levels. The bars represent the means, and the error bars the standard deviations.

**Figure S3: Exposure to Bleomycin results in consistent levels of SOS induction across a concentration range normalized normalized to pitB-GFP.** (a) Magnitude of SOS induction when strains are exposed to a range of Ciprofloxacin concentrations averaged across two biological replicates. (b) Magnitude of SOS induction when strains are exposed to a range of Bleomycin concentrations averaged across two biological replicates with each black dot representing a replicate. * denotes MIC. Error bars represent SD.

**Figure S4: Solvent used to dissolve bioactive compounds have no effect on induction.** (a) Phage quantification of HK97 lysogen alone and challenged with DMSO or PCR H_2_O averaged across three biological replicates. (b) Phage quantification of HK97 Δ*recA* lysogen alone and challenged with DMSO or PCR H_2_O averaged across three biological replicates with each black dot representing a replicate. Error bars represent SD.

**Table S1: MIC of validated prophage inducers.**

| **Bioactive Compound** | **MIC** |
| --- | --- |
| Mitomycin C | 0.125 μg/mL |
| Ciprofloxacin | 0.063 μg/mL |
| Bleomycin | 6.25 μg/mL |
| Harmane | 100 μg/mL |
| Fluoxetine | 100 μg/mL |

**Table S2: Z' Factor Determination for HTS Assay Validation.**

| **Time point** | ***E. coli* K12** HER1382 **+ 1% DMSO** | ***E. coli*** K12 HER1382 **HK97 lysogen + 1% DMSO** |
| --- | --- | --- |
| 5 hrs | 0.7868 | 0.6585 |
| 5.5 hrs | 0.8130 | 0.6681 |
| 6 hrs | 0.8085 | 0.6823 |

**Table S3: List of primary bioactive compounds hits.** Organized from greatest effect on OD to least effect. Cells highlighted in yellow indicate validated prophage inducers. Cells highlighted in grey indicate compounds unable to be verified as prophage inducers.

|  | **Bioactive Compound** |
| --- | --- |
| **1** | Meptazinol hydrochloride |
| **2** | Desoxymetasone |
| **3** | Alanyl-dl-leucine |
| **4** | Valproic acid sodium |
| **5** | Valacyclovir Hydrochloride |
| **6** | Harmane |
| **7** | Epirubicin Hydrochloride |
| **8** | Tolazoline Hydrochloride |
| **9** | Chlorophyllide Cu Complex Na salt |
| **10** | 1,3-Dipropyl-8-p-sulfophenylxanthine |
| **11** | Thiothixene hydrochloride |
| **12** | Lercanidipine hydrochloride hemihydrate |
| **13** | Olomoucine |
| **14** | Chlorambucil |
| **15** | Tramadol Hydrochloride |
| **16** | Oxantel pamoate |
| **17** | Lomefloxacin hydrochloride |
| **18** | AL-8810 |
| **19** | Docosanol |
| **20** | Imiquimod |
| **21** | Betulinic acid |
| **22** | Lobaric acid |
| **23** | Ethyl Everninate |
| **24** | Gibberellic acid |
| **25** | Willardiine |
| **26** | Salidroside |
| **27** | Liquiritigenin Dimethyl Ether |
| **28** | Lycorine |
| **29** | Ofloxacin |
| **30** | Astaxanthin |
| **31** | Isoquinoline hydrochloride |
| **32** | Berberine |
| **33** | Nystatine |
| **34** | Fluoxetine hydrochloride |
| **35** | Bleomycin B2 |
| **36** | Rolitetracycline |
| **37** | Cefepime hydrochloride |
